# A Validation of the Spectral Power Clustering Technique (SPCT) by Using a Rogowski Coil in Partial Discharge Measurements

**DOI:** 10.3390/s151025898

**Published:** 2015-10-13

**Authors:** Jorge Alfredo Ardila-Rey, Ricardo Albarracín, Fernando Álvarez, Aldo Barrueto

**Affiliations:** 1 Departamento de Ingeniería Eléctrica, Universidad Técnica Federico Santa María, Av. Vicuña Mackenna 3939, Santiago de Chile 8940000, Chile; E-Mail: aldo.barrueto@usm.cl; 2 Generation and Distribution Network Area. Dept. Electrical Engineering. Innovation, Technology and R&D, Boslan S.A. Consulting and Engineering, Calle de la Isla Sicilia 1, Madrid 28034, Spain; E-Mail: rasbarracin@gmail.com; 3 Departamento de Ingeniería Eléctrica, Universidad Politécnica de Madrid, Ronda de Valencia 3, Madrid 28012, Spain; E-Mail: fernando.alvarez@upm.es

**Keywords:** partial discharges (PD), Rogowski coil, wideband PD measurements, clustering techniques, condition monitoring, electrical insulation condition, on-line PD measurements, pattern recognition, signal processing

## Abstract

Both in industrial as in controlled environments, such as high-voltage laboratories, pulses from multiple sources, including partial discharges (PD) and electrical noise can be superimposed. These circumstances can modify and alter the results of PD measurements and, what is more, they can lead to misinterpretation. The spectral power clustering technique (SPCT) allows separating PD sources and electrical noise through the two-dimensional representation (power ratio map or PR map) of the relative spectral power in two intervals, high and low frequency, calculated for each pulse captured with broadband sensors. This method allows to clearly distinguishing each of the effects of noise and PD, making it easy discrimination of all sources. In this paper, the separation ability of the SPCT clustering technique when using a Rogowski coil for PD measurements is evaluated. Different parameters were studied in order to establish which of them could help for improving the manual selection of the separation intervals, thus enabling a better separation of clusters. The signal processing can be performed during the measurements or in a further analysis.

## 1. Introduction

The electrical generation, transmission and, even distribution infrastructures require large financial investments, so their long-term profitability must be optimized. In this context, there has been a growing interest on the one hand, to reduce maintenance cycles applied to electrical machinery and power cables when they are very aged and secondly, to adequately plan their replacement when its operation becomes unreliable [1].

For these reasons, it is assumed that electrical equipment must be replaced every certain period of time, close to 30–40 years [2,3], and that the maintenance cycles must be fixed in advance (preventive maintenance). However, the progress made in basic electrical insulation research and the increase in the availability of historical failure data allows choosing new maintenance strategies. Through these strategies, it is possible to know the operation condition of the electrical assets by performing in service (on-line) measurements in high-voltage installations. This procedure extends the lifespan of the equipment, as well as their periods of scheduled maintenance [4]. Thus, the lack of investment, that is often required in the replacement of equipment [3], could be compensated with the implementation of a proper Condition-Based Maintenance (CBM) program [5]. For these reasons, PD measurement has become a major diagnostic method used in the maintenance of electrical installations, in order to establish the degradation of the insulation systems, since its lifetime is determined by the degree of degradation present [6].

In measurements made on-site or even in controlled environments, such as high-voltage laboratories, the pulses from multiple PD sources and electrical noise may be overlapped, thus creating complex phase-resolved PD (PRPD) patterns. In some cases, the noise signals can have magnitudes greater than the PD pulses, so the raising the trigger level of the acquisition systems is not a valid PD separation technique. This problem has increased due to the growing use of electronic power converters in electrical systems (variable frequency drives, power supplies switching, rectifiers, inverters, converters, *etc.*). Consequently, source separation has become a fundamental requirement in obtaining an effective diagnostic, such that avoid erroneous assessments in the equipment or system insulation.

Many modern measuring instruments are equipped with pulse classification tools that are based on characterization of the waveforms of the acquired pulses, inasmuch as noise and PD pulses generated by different sources present different shapes.

The classification procedures require broadband sensors capable of detecting ranges up to tens of MHz [7,8]. Commonly, inductive sensors are used for PD measurements. These sensors are capable of measuring according to the standard detection circuits. The most widely used are the high-frequency current transformers (HFCT), inductive loop sensors (ILS) and the Rogowski coils (RC) [9,10,11,12,13,14]. Recent studies have shown that the SPCT applied to the pulses obtained with HFCT and ILS sensors measuring in different test objects, have been successfully characterized and its effectiveness to separate different PD sources and electrical noise has been proven, even when these sources are simultaneously active [15].

In this paper, the ability of clustering by applying the SPCT to PD pulses and electrical noise acquired with a RC for various test objects in two different environments is evaluated. The aim of the paper is to show the benefits of SPTC technique, even when sensors with a poor transfer impedance are used. To this end, the RC was used, since it has an air-core of non-magnetic material, which provides linearity and low self-inductance [13]. This type of core allows designing sensors with lower weights, cheap and more flexible, allowing more applicability and easy-to-use.

Additionally, the behaviour of the different frequency bands is studied when multiple sources are present during the acquisition. This is done, in order to establish some important indicators, that allow the operator of the classification tool (based on SPCT), to evaluate whether the selected frequency ranges are the most appropriate, when it comes to separate the different sources that may be present during the measurement.

## 2. Rogowski Coil

**Figure 1:**
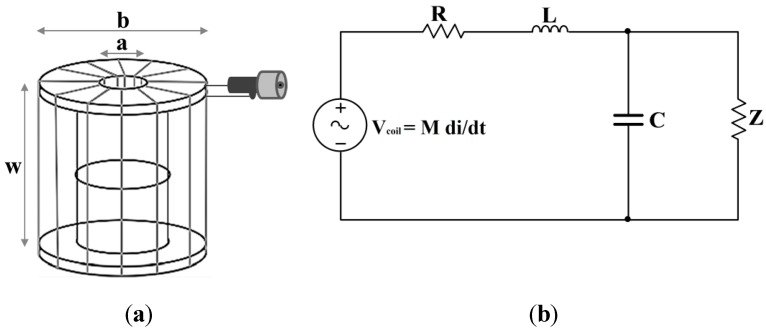
(**a**) Rogowski Coil; and (**b**) its electric equivalent circuit.

The RC, as well as the different inductive sensors commonly used for PD detection (HFCT or ILS) [9,15], operates on the basic principle of the Faraday’s Law and can be applied to measure PD [16]. Accordingly, the air-core coil is placed around the conductors through which the current pulses associated to PD and electrical noise can be propagated. This variable current produces a magnetic field, which links the secondary of the coil and induces a voltage directly proportional to the rate of change of the current in the conductor and the mutual inductance between the coil and the conductor. The RC designed and used in this paper, is based on a toroidal transformer with an air-core of transversal rectangular section, made of 12 identical turns as on the geometry indicated in Figure 1a. This configuration is modelled with the equivalent circuit shown in Figure 1b, with the induced voltage represented by the source voltage *V_coil_* and the electrical effects of the winding represented by *R*, *L* and *C* parameters, which correspond with the resistance, self-inductance of the winding cable and capacitance between the coil turns and the return cable, respectively. The geometric and electrical parameters for this design are indicated in Table 1.

**Table 1:** Parameters of the Rogowski Coil.

a (cm)	b (cm)	w (cm)	M (nH)	R (Ω)	L (nH)	Z (Ω)	C (pF)
0.5	3	2	86	0.038	1032	50	18.1

Considering the equivalent circuit and the electric characteristics of this sensor, its transfer function is defined by Equation (1):

(1) $$\frac{V_{out}\left(s\right)}{I\left(s\right)}=\frac{4.30\cdot 10^{-6}s\;}{9.38\cdot 10^{-16}s^{2}+1.032\cdot 10^{-6}s+50.038}$$

**Figure 2:**
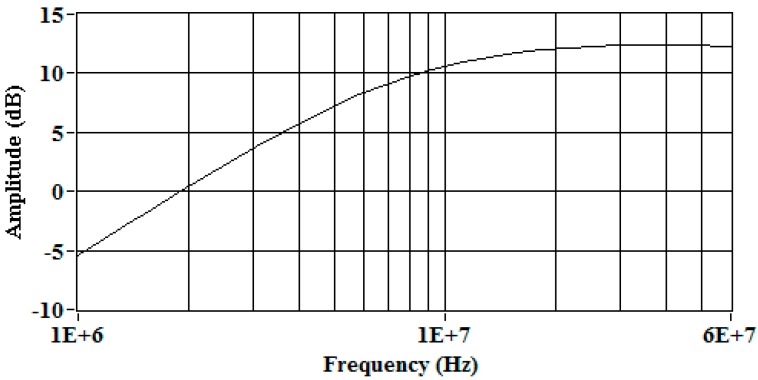
Frequency response of the Rogowski coil.

It is important to indicate that Z is usually 50 Ω and represents the input impedance of the measuring instrument, where the sensor is connected. More details about this type of sensor and the calculation of the electric parameters can be found in [12,13]. Finally, the frequency response for this sensor, calculated from Equation (1), is shown in Figure 2. The results indicate that the coil has a derivative behaviour up to 9 MHz approximately, and then the output signal turns into to a voltage proportional to the current. Moreover, the sensitivity is around 12 dB. The frequency analysis for this sensor is presented up to 60 MHz, since the observation of the average spectra for the signals measured in the experiments presented in the following sections, led to the conclusion that the power above 60 MHz was very low.

## 3. Experimental Setup and PD Sources Separation

### 3.1. Experimental Setup

Due to the importance of PD phenomenon to estimate the insulation lifetime, a procedure to measure PD including the circuits implemented for their detection is detailed in the standard IEC 60,270 [6]. Although the measured PD signals are different to the original signals originated in the PD sources, due to the attenuation and dispersion effects until the sensor captures them, much information of the pulse shape to distinguish their source type (internal, surface or corona) can be obtained [17,18,19].

**Figure 3:**
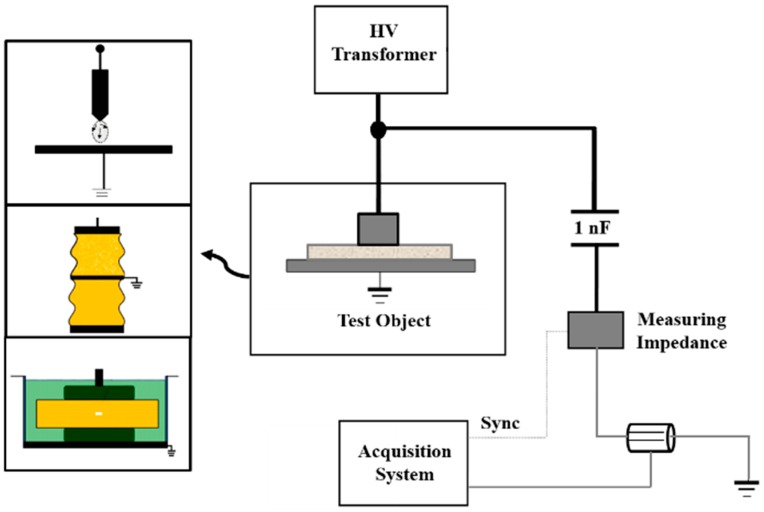
Experimental setup for PD measurements.

Accordingly, in this paper all data analysed have been collected experimentally with an indirect detection circuit based on the standard IEC 60,270. The circuit consists of a 750 VA transformer that provides high-voltage to several test objects, where PD are created. A capacitive divider with a high-voltage capacitor (1 nF), connected in series with a measuring impedance, provides a path for the high-frequency currents generated by the PD pulses, see Figure 3. Pulses flowing through the capacitive path are measured using the Rogowski sensor presented in Section 2. The measuring impedance gives the synchronization signal from the grid frequency to the measuring instrument, so PD pulses can be plotted in conventional PRPD patterns.

A NI-PXI-5124 digitizer was programmed to get the information from each experiment. The technical characteristics of this digitizer are 200 MS/s of sampling rate, 12 bits of vertical resolution and 150 MHz of bandwidth. The channel 0 was used for the 50 Hz reference voltage signal measurement and the channel 1 was used to get the waveform of the high-frequency pulses. This acquisition system acquires data each 20 ms (network cycle). These data are divided in time windows of 1 μs or 4 μs, depending on the duration of the PD pulses. Only the data that have peaks higher than the trigger level in the time windows are considered; the rest are discarded. Each signal measured is represented by 200 or 800 samples corresponding to the time window widths of 1 μs or 4 μs and is stored in vectors, in order to calculate contents in frequency up to 100 MHz. As shown in Figure 3, some basic PD sources such as corona effect, a surface defect and an internal defect were created through some simple test objects (see [10] for more details):

- Corona effect: point-plane experimental test object. A 0.5 mm thick needle was placed above a metallic ground plane. The distance between the needle and the plane was adjusted to 1 cm.
- Surface defect: Contaminated ceramic bushing. A 15 kV ceramic bushing was contaminated by spraying a solution of salt in water to create ionization paths along the surface. In order to avoid unstable PD activity, the measurements are carried out once moisture has been disappeared.
- Internal defect: Insulating sheets immersed in mineral oil. This setup was designed to generate internal discharges and consists of eleven insulating sheets of NOMEX paper (polyimide 0.35 mm thick film). The central paper sheet was pierced with a needle (1 mm in diameter) to create an air void inside this dielectric.

As it will be indicated later in the experimental results, the experimental setup was implemented identically in two different high-voltage laboratories, one that is completely shielded and another that is unshielded. This was done in order to characterize the ability of the PR maps to separate PD sources in two different environments measuring with the RC sensor. In the first laboratory, controlled, the noise signals present a low magnitude and in the second one, less controlled, the noise signals present similar characteristics to those found in industrial environments: high-levels of amplitude and high-spectral variability.

### 3.2. Spectral Power Clustering Technique (SPCT)

Separation and identification of PD sources are stages that must be approached sequentially, due to separation is a prerequisite fundamental and obligatory for a successful and accurate identification.

When PD measurements are carried out with inductive sensors, such as the RC, the waveform of the carrying currents sensed as a result of PD activity cannot be universally identified with a particular type of PD source (corona, surface or internal), due to the stochastic behaviour of PD phenomena and due to the distortion caused in the pulse transmission from the source to the measuring point and in the coupling system itself. However, PRPD patterns allow to successful identifying PD sources [19]. Therefore, a generic solution widely used in most PD measuring instruments, is based on the analysis of the entire PRPD pattern containing all sources measured and on the separation of this pattern into sub-PRPD patterns, each corresponding to a specific source. The separation is accomplished by assuming that each PD source exhibits similar waveforms, while the signals produced by different sources are different. Following this premise, this paper attempts to prove that the SPCT allows separating different PD and noise sources, mapping for each of the measured signals the value of the relative spectral power calculated for two intervals: PRL (power ratio for low-frequencies) and PRH (power ratio for high-frequencies).

**Figure 4:**
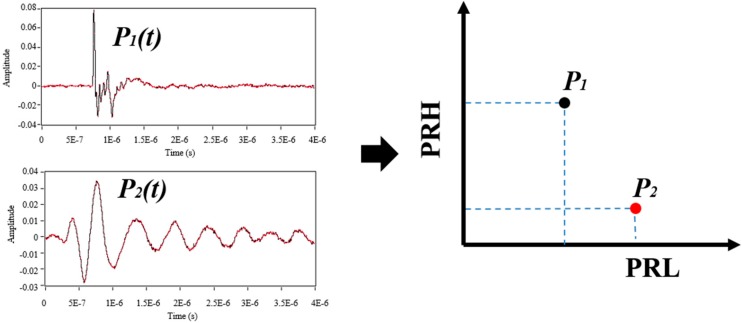
Example of PR map for two pulse sources (PD and noise).

In this approach, the pulses are analysed in the frequency domain therefore, the fast Fourier transform is applied to each detected pulse, obtaining its spectral magnitude distribution *s*(*f*) [10]. Then, the spectral power of each pulse is calculated in two frequency intervals, [*f_1L_, f_2L_*] and [*f_1H_, f_2H_*]. Since the total spectral power or amplitude of the signals may influence the pulse characterization, these spectral powers are divided into the overall spectral power calculated up to the maximum analysed frequency *f_t_*. The obtained quantities are defined as power ratios (%), one for the higher frequency interval, PRH, and another for the lower frequency interval, PRL, as shown in Equations (2) and (3). These two parameters are represented in a two dimensional map, where each pulse source showed a different cloud of points (clusters) with different positions, (see Figure 4).

For all measurements, the frequency analysis was made up to 100 MHz, however, the observation of the average spectra for all the experiments led to the conclusion that the power above 60 MHz was very low, so this last value was used as *f_t_*.

Thus, the power ratio for low-frequencies (PRL) and the power ratio for high-frequencies (PRH) are calculated as follows:

(2) $$PRL=\frac{\sum_{f_{1L}}^{{\text{f}}_{2\text{L}}}{\left|s\left(f\right)\right|}^{2}}{\sum_{0}^{f_{t}}{\left|s\left(f\right)\right|}^{2}}\cdot 100$$

(3) $$PRH=\frac{\sum_{f_{1H}}^{f_{2H}}{\left|s\left(f\right)\right|}^{2}}{\sum_{0}^{f_{t}}{\left|s\left(f\right)\right|}^{2}}\cdot 100$$

For all measurements presented in this paper, the frequencies for the PRL and PRH calculation were set to: *f_1L_* = 10 MHz, *f_2L_* = *f_1H_* = 30 MHz, *f_2H_* = 50 MHz, *f_T_* = 60 MHz. The interval, [0, 10] MHz was not taken into account in the analysis due to the derivative behaviour of the sensor in this frequency band. In addition, these intervals are the same as those used in [15] for the initial analysis of clusters.

## 4. Experimental Results

In the first part of the experimental results, the measurements were carry out using the indirect circuit described in Section 3.1, but housed in the shielded high-voltage laboratory. The sources were initially characterized individually and were measured in the following way:

1. The noise signals present in the laboratory were registered, by performing measurements with a low trigger level and by applying a low-voltage to the test object.
2. Then, the voltage and the trigger levels were increased until a stable PD activity noise-free was found for each of the PD sources.

To obtain statistically significant results and guarantee the reliability of the phenomenon observed, the number of pulses acquired by the measuring instrument for each of these measurements must be high and was set in 1500. Then, the clusters associated with PD and noise were characterized again, but when the different type of PD sources were simultaneously emitting. In this case, the measurements were performed for a high-voltage level, but with a reduced trigger level, in order to enable the acquisition of PD and noise simultaneously. For this last measurement, over 3000 pulses were acquired, since it was present more than one type of pulse sources.

For each experiment, the PR maps and the average spectral power of the pulses are described. Additionally, the dispersion obtained in PRL and PRH parameters was compared for each of the clusters (PD or noise), in order to evaluate which type of source has greater dispersion when a RC sensor is used. This information is of great interest because, as described in [15], the location and shape adopted by clusters in the PR maps depends, not only on the frequency intervals, on the equivalent capacitance of the test object, and on the pulse nature, but also on the type of sensor used. Therefore, the more homogeneous are the clusters associated with a particular source the better is its characterization when different sources are detected, especially if there is a clear separation between them.

In the second part of the experimental results, the measurements were made using the same indirect measurement circuit but in this case, this was housed in the unshielded laboratory. Again, the clusters associated to PD and electrical noise were characterized in these tests, when different types of sources were present simultaneously.

### 4.1. Experimental Measurements Performed in the Shielded Laboratory

The measurements described below, were made, in order to obtain a controlled environment to minimize the influence of electrical noise, and to facilitate the characterization of the dispersion of the clusters of pulses for each type of PD source. However, as it will be indicated in Section 4.1.3, it is not possible to completely minimize the influence of noise during the acquisitions, especially when the measurements are performed with a RC sensor, whose signal-to-noise ratio (SNR) is low compared to other types of inductive sensors [20,21,22].

#### 4.1.1. Noise Characterization

**Figure 5:**
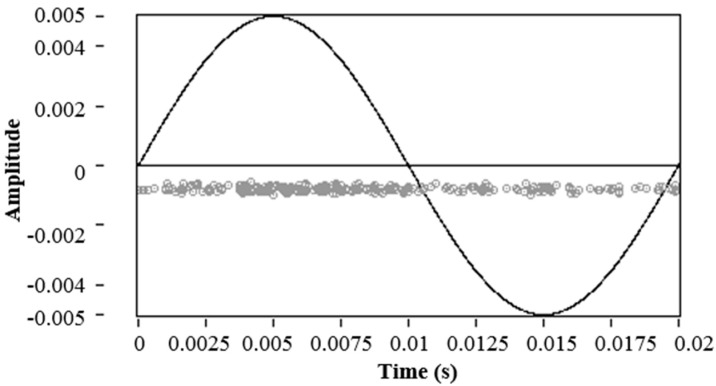
PRPD for noise acquisition in the point-plane experimental test object.

In order to characterize the noise sources in the three test objects described in the experimental setup, a low-voltage (800 V) level was applied to each test object. Additionally, the trigger level in the acquisition system was set at low level (0.4 mV). This procedure ensures that the pulses obtained with the RC correspond to sources of electrical noise and not to PD sources, since the voltage level is very low to start PD activity. It can be confirmed, for the data acquired in the case of the point-plane experimental test object (see Figure 5), that the PRPD pattern obtained is the typical pattern of the electrical noise (uncorrelated in phase). In this case, the maximum noise levels found were close to 1 mV.

**Figure 6:**
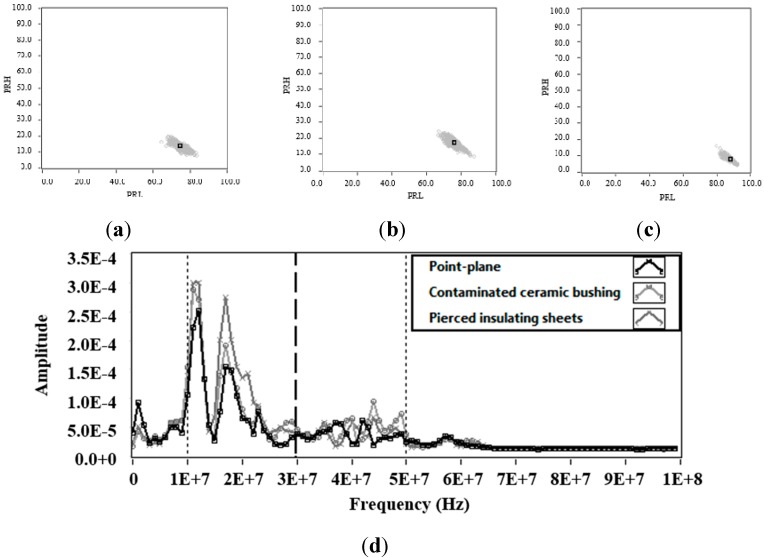
Noise acquisition 800 V. (**a**) Power ratio map for noise signals in the point-plane experimental test object; (**b**) Power ratio map for noise signals in a contaminated ceramic bushing; (**c**) Power ratio map for noise signals in pierced insulating sheets; (**d**) Average spectral power of the signals obtained with the three test objects.

Figure 6a–c show the PR maps for the signals associated with electrical noise that were obtained for each test object. In all measurements, the noise was clearly characterized as a cloud of points in the lower right part of the map. This position is coherent with Figure 6d corresponding to the average spectral power to each of the signals, where the spectral power content in the interval [10, 30] MHz (PRL), is higher than the obtained in the interval [30, 50] MHz (PRH). The high spectral power obtained for the interval PRH in each of the measurements of noise is due to the presence of two peaks of power around 12 MHz and 18 MHz. These characteristics are typical of conventional noisy environment, whose behaviour is narrow-band. In all noise measurements that were made for each test objects the average spectral power has the same behaviour in PRL and PRH, see Figure 6d.

In order to evaluate the statistical dispersion of the clusters obtained in each experiment, the standard deviation for PRL and PRH was calculated. The standard deviation was obtained from the centroid for each cluster according to the following mathematical expression:

(4) $$\sigma_{PRL}=\sqrt{\overset{n}{\underset{i=1}{\sum }}\frac{{(X_{i}-CX)\;}^{2}}{n}}$$

(5) $$\sigma_{PRH}=\sqrt{\overset{n}{\underset{i=1}{\sum }}\frac{{(Y_{i}-CY)\;}^{2}}{n}}$$

where *n* is the number of points in each clusters, *X_i_* and *Y_i_* is the position (PRL-PRH) of each point *i* in the PR map and (*CX*, *CY*) is the centroid of the cluster, which is obtained according to described in [15]. Table 2 summarizes the results of the dispersion obtained for each cluster associated with electrical noise.

**Table 2:** Results obtained of the standard deviation for each cluster associated with electrical noise.

Indicator	Point-Plane Experimental Specimen	Contaminated Ceramic Bushing	Pierced Insulating Sheets
*σ_PRL_*	3.15	3.10	3.02
*σ_PRH_*	2.23	2.52	2.35
*σ_PRL_/σ_PRH_*	1.41	1.23	1.28

Analysing the results in Table 2, the dispersion in PRL and PRH for each of the clusters has very similar values. When comparing the dispersion between PRL and PRH for each cluster, it is observed that the dispersion in PRL is higher than that obtained by PRH, this last is most notable in the case of the point-plane experimental test object, where the *σ_PRL_/σ_PRH_* ratio is higher than for other test objects (1.41). This ratio will allow us to identify during the measurements in which axis of the PR map, is more dispersed one cluster, *i.e*. if *σ_PRL_/σ_PRH_* > 1; PRL has a greater dispersion, otherwise if *σ_PRL_/σ_PRH_* < 1 this means that PRH has the greater dispersion.

From the point of view of source separation, an ideal cluster is the one that has a ratio *σ_PRL_/σ_PRH_* ≈ 1 and a high homogeneity (low dispersion in PRL and PRH). Therefore, if the clusters located on the classification map are obtained with these characteristics (*i.e*., *σ_PRL_/σ_PRH_* ≈ 1, low *σ_PRL_* and low *σ_PRH_*), it will be obtained a very homogeneous clouds of points that facilitates the application of any method of clusters identification (K-means, K-medians, Gaussian, *etc*.), after the application of the SPCT, so a better separation and identification of points associated with each cluster is achieved when multiple sources are present.

It is important, for the operator of the classification tool, to consider this information once each of the sources in the classification map have been characterized, as this can help to verify if the separation intervals manually selected should be modified slightly or completely changed in order to enhance the clusters separation. This will facilitate, in a later stage, the sources identification process, that can be performed through visual inspections or applying automatic identification algorithms. Furthermore, it must be emphasized that this information is only useful once the intervals of separation have been selected, because these indicators alone cannot estimate the frequency bands where the separation intervals should be located. They only allow assessing homogeneity, dispersion and shape of the clusters for each of the previously demarcated intervals.

However, in Section 4.1.3, an additional graphic indicator is presented. This is based on the variability of the spectral power of the captured signals, which does allow identifying areas of interest where the user must locate the separation intervals, in order to obtain an initial characterization which may, or may not, be improved by modifying slightly the position of the separation intervals or evaluating the dispersion in PRL (*σ_PRL_*), PRH (*σ_PRH_*) and its ratio (*σ_PRL_/σ_PRH_*), for each case, up to a better characterization of each source.

#### 4.1.2. Partial Discharge Source Characterization

**Figure 7:**
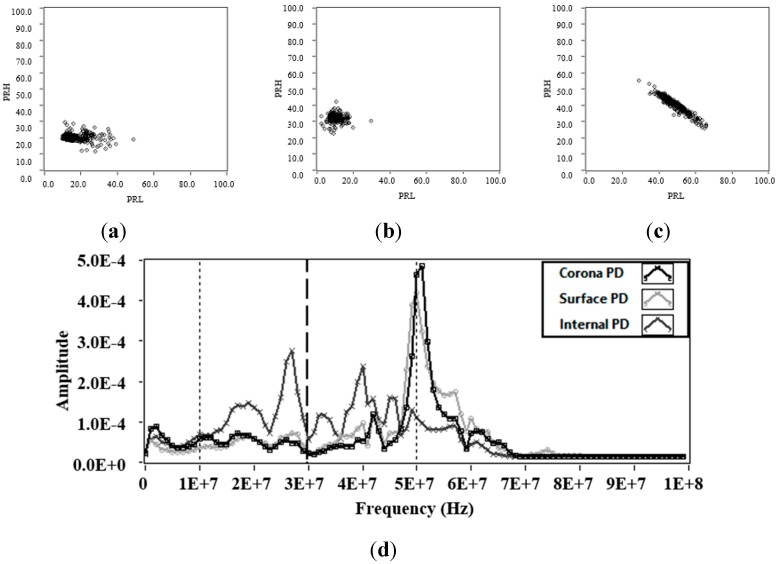
Partial discharges (**a**) Power ratio map for PD in point-plane experimental test object; (**b**) Power ratio map for PD in contaminated ceramic bushing; (**c**) Power ratio map for PD in pierced insulating sheets; (**d**) Average spectral power of the PD signals obtained with the three test objects.

In order to find stable PD activity and to avoid the acquisition of noise signals, the following measurements were made for high-voltages applied and high-trigger levels (1.2 mV). Figure 7a–d represent the PR maps and the average spectral power of the signals measured by applying 5 kV to the point-plane experimental test object, 8.3 kV to the ceramic bushing and 9 kV to the pierced insulating sheets respectively.

Figure 7d shows that the spectral power components detected by the RC in the intervals [10, 30] MHz and [30, 50] MHz are higher for the pulses associated to internal PD (PD in the pierced insulating sheets), this can be confirmed in Figure 7c, where the position of the cluster with respect to the PRL and PRH axis is higher than that obtained with the clusters of corona and surface PD. Figure 7d also shows, that for the pulses associated to corona and surface PD, the spectral power detected is very similar in the interval [10, 30] MHz, while in the interval [30, 50] MHz the spectral power is slightly higher for surface PD, which is consistent with the position of the clusters in both classification maps, see Figure 7a (corona PD) and b (surface PD).

Regarding the dispersion of the clusters associated to PD (see Table 3), the results indicate that the internal PD clusters present the higher spectral power dispersion, both in PRL and PRH (5.41 and 4.40, respectively). On the contrary, the dispersion was considerably low for the surface PD clusters, thus together with the fact that the *σ_PRL_/σ_PRH_* ratio is close to 1, makes the cluster for this type of source be very homogeneous and with low dispersion. The latter is particularly important because, as will be shown in the next section, a high homogeneity in clusters facilitates the separation task and their subsequent display of the PRPD pattern if there are present several sources acting simultaneously.

In these measurements, it is noted that the dispersion in PRL continues to be higher than that obtained in PRH. Again, the *σ_PRL_/σ_PRH_* ratio is much greater for the PD obtained for the point-plane experimental test object (3.18).

**Table 3:** Results obtained of the standard deviation for each cluster associated with PD.

Indicator	Corona PD	Surface PD	Internal PD
*σ_PRL_*	5.12	2.11	5.51
*σ_PRH_*	1.61	1.63	4.40
*σ_PRL_/σ_PRH_*	3.18	1.29	1.25

#### 4.1.3. PD and Noise Characterization

**Figure 8:**
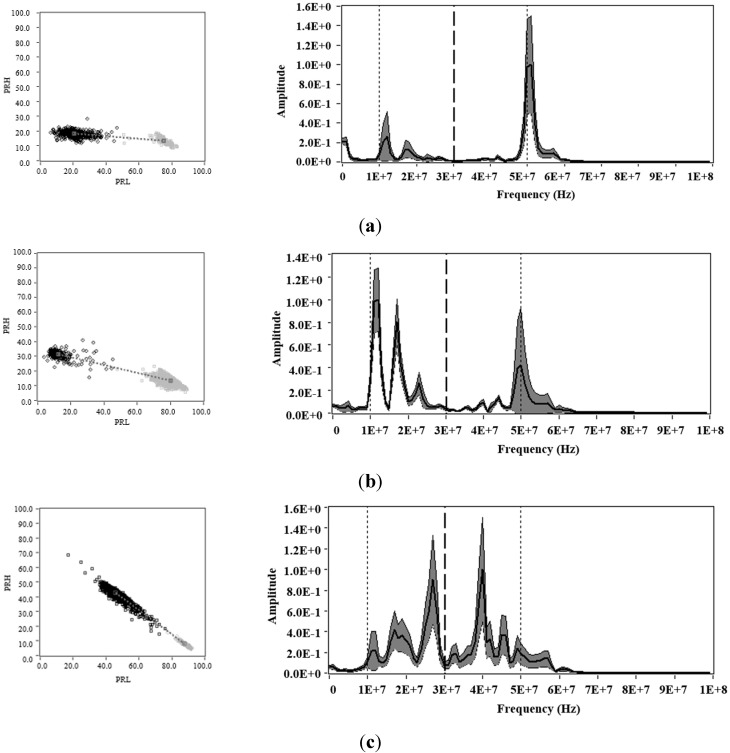
Power ratio maps for PD (black cluster) and noise (grey cluster), **left**: Average power spectrum densities, **right**: Obtained for (**a**) corona PD and noise; (**b**) surface PD and noise and; (**c**) internal PD and noise.

In this section, measurements were performed for each of the test objects with the same voltage level used in the previous section, but with a reduced trigger level (0.4 mV). This was made to enable the acquisition of PD and noise simultaneously. Figure 8 (left), shows the PR map with the clusters associated to PD (black cluster) and noise (grey cluster). Figure 8 (right) shows the average power spectrum densities, for each of PD and noise pulses that are represented on the PR map and that are acquired for each test object.

Considering that for this experiment, there are two sources simultaneously acting, the average power spectrum density is presented in order to see if in the selected intervals (*f_1L_* = 10 MHz, *f_2L_* = *f_1H_* = 30 MHz, *f_2H_* = 50 MHz, *f_T_* = 60 MHz) are included the bands where greater variability of spectral power is presented. If these are included, a clear separation of sources (PD and noise) could be achieved in the PR maps, since for these bands, the captured pulses have less similarity. Otherwise, if the bands with less variability of spectral power are selected, the clusters could be overlapped and the separation of sources could result more difficult. The average of the spectrums is plotted in central thick line; the shaded area corresponds to the area at one standard deviation of the mean that was obtained for the pulses in each measurement.

For each of the experiments, the *K-means* algorithm [23] has been used to identify the clusters and its centroids after applying the SPCT to the pulses measured.

As expected, the clusters associated to PD and noise tend to take similar positions as those observed in Figure 6 (only noise) and Figure 7 (only PD). However, the position of the clusters no longer matches with the average spectral power obtained (central thick line), since the spectral content dependent on the spectral power of both types of sources (PD and noise). Therefore, the spectral components will be affected by the two sources acting simultaneously during the acquisition. On the other hand, as was described above, the standard deviation (shaded area) helps to indicate the frequency bands where there is greater statistical variability. With this information, the user can select or modify the frequency bands, in order to improve the separation of sources. Accordingly, for this experiment it is observed that both PRL and PRH include some frequency bands where the standard deviation of the frequency spectra was high. This allows the identification in the three cases, the presence of the two clusters, one associated with PD and other with electrical noise.

**Figure 9:**
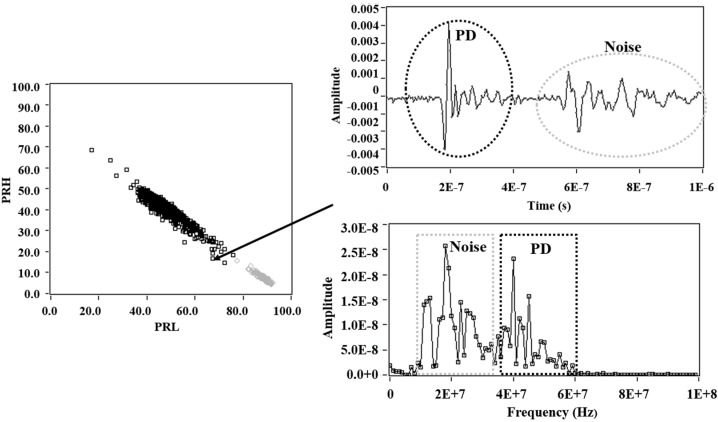
Example of a pulse formed by components of PD and noise.

Note that, in these intervals, also the frequency bands where the standard deviation is minimal were included; therefore, this separation can be improved if only the bands with the higher standard deviation are selected. When evaluating the dispersion values in PRL and PRH, which are summarized in Table 4, it is found that the dispersion in each of the clusters associated with PD was increased. This occurs due to the low trigger level during the acquisition, so the noise pulses can be added to the PD pulses, generating signals with combined spectral power components that cause an increase of the dispersion in the clouds of points. For example, in the case of internal PD and noise in Figure 8c, that can be considered the most extreme case for having the largest dispersion in PRL and PRH, if it is represented one of the points of the PR map that is located between the two cluster (see Figure 9), clearly it is observed that the spectral power content of this pulse is formed by components of both sources, see Figure 6d and Figure 7d. Due to this, the pulses tend to be located in an intermediate region of the two clusters (critical zone), hindering the separation process and the subsequent identification of the sources.

**Table 4:** Results obtained for the standard deviation and its increase in each cluster associated with PD and electrical noise.

Indicator	Corona PD and Noise			Surface PD and Noise			Internal PD and Noise		
	PD	Increase (%)	Noise	PD	Increase (%)	Noise	PD	Increase (%)	Noise
*σ_PRL_*	6.69	**30.66**	3.10	6.02	**185.30**	4.28	6.71	**21.77**	2.42
*σ_PRH_*	1.97	**22.36**	2.27	2.71	**66.25**	2.68	5.19	**17.95**	1.88
*σ_PRL_/σ_PRH_*	3.39	**6.60**	1.36	2.22	**72.02**	1.59	1.29	**32.00**	1.28

However, despite this increase in *σ_PRL_* and *σ_PRH_* for each of the clusters associated with PD due to the presence of an additional source of electrical noise, it has been possible to show that the RC allows to characterize adequately in different areas of the map both types of sources by applying the SPCT.

### 4.2. Experimental Measurements Performed in the Unshielded Laboratory

In order to evaluate the performance of the RC in a less controlled environment, where the noise present has completely different characteristics in time and frequency to those found in the previous experiments, new measurements were carried out in a second high-voltage laboratory. In this second emplacement, there is not any type of shielding that can minimize the presence of external noise sources generated. Additionally, the laboratory is in an area of industrial activity (surrounded by industrial facilities), where the noise level can vary depending on the external activity during the measurement process.

On the other hand, the experimental setup used in this section was prepared to simultaneously generate three different sources: one associated with corona another to internal PD and the last one associated with electrical noise. Thus, the pulse sources were measured with the RC working in a very similar environment to that found in on-site measurements, where most of times it is necessary to detect and separate simultaneous PD and noise sources in order to identify the insulation defects involved.

For this purpose, the tests objects used for internal and corona PD were modified to obtain a stable PD activity for the same voltage level on both test objects (5.2 kV). In this case, the separation from the needle to the ground in the point-plane configuration was 1.5 cm. For the internal defect, the insulation system was composed by eleven insulating sheets of NOMEX where the five central sheets were pierced. Then, an air cylinder with 5 × 0.35 mm in height inside the solid material is obtained. For this experiment, the trigger level was set at 1.3 mV (low), because the maximum noise levels found were close to 2.1 mV, this level of noise is greater than that in the laboratory shielded (1 mV).

**Figure 10:**
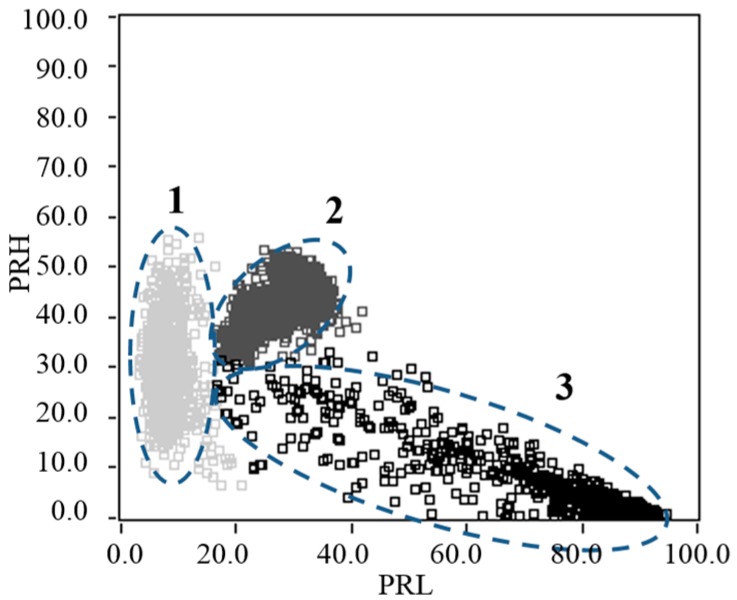
PR map obtained for noise (cluster 1), corona PD (cluster 2) and internal PD (cluster 3).

Finally, both test objects were electrically connected in parallel and subjected to 5.2 kV, measuring thousands of PD and noise pulses waveforms. The resulting PR map for this experiment, maintaining the same intervals of separation as previously (*f_1L_* = 10 MHz, *f_2L_* = *f_1H_* = 30 MHz, *f_2H_* = 50 MHz, *f_T_* = 60 MHz) is presented in Figure 10. For this case, three different clouds of points can be easily selected (since they are clearly separated) to identify the PD source type through the PRPD patterns.

The position of the cluster associated with the new source of electrical noise, indicates that the spectral power in the range [10, 30] MHz is lower than that obtained in the previous experiments, in which the source of noise had a high spectral power for the same interval. As for the values of dispersion in PRL and PRH shown in Table 5, it is seen that the dispersion in PRL is 2.62, lower than previously values previously obtained in Table 2 for the noise in all the test objects. Contrary to this, the dispersion in PRH is increased almost 292% for the point-plane experimental test object, 247% for the contaminated ceramic bushing and 272% for the pierced insulating sheets; which is consistent with the form taken by this cluster on the PR map and (see Figure 10). In addition, the *σ_PRL_/σ_PRH_* ratio for this new source happened to be well below 1 (0.30).

**Table 5:** Results obtained of the standard deviation for each cluster associated with PD and noise.

Indicator	Noise	Corona PD	Internal PD
*σ_PRL_*	2.62	4.08	11.51
*σ_PRH_*	8.75	3.68	4.45
*σ_PRL_/σ_PRH_*	0.30	1.10	2.58

For the cluster associated with corona PD pulses, it was also observed a variation in the shape and the position on the PR map, which differs greatly from previous experiments. The values shown in Table 5 indicate that the dispersion in PRL and PRH suffered significant changes (*σ_PRH_* increases and *σ_PRL_* decreases). For this cluster, the new relation *σ_PRL_/σ_PRH_* was 1.10. A value close to unit means that the cluster takes a more “symmetric” shape. As mentioned throughout this paper, when it has this kind of geometries or forms in the clusters it facilitates the identification process when the operator have to select the cluster to be represented its respective PRPD pattern, improving the process of identifying the type of source.

Finally, the cluster associated with internal PD also presents great changes, both in position and in shape, according to its PR map characterization. The most notable change, in terms of dispersion, is observed for PRL, which it is increased by almost 108% over the value of PRL obtained in previous experiments (see Table 3), this is easily seen in the PR map in Figure 10, where the cluster occupies a large map space due to its lack of homogeneity in this axis. In this case, the relationship *σ_PRL_/σ_PRH_* (2.58) indicates an increase of almost 106% compared to the values previously obtained when the size of the vacuole was lower.

These results confirm those described in [10,15], where is disclosed that any change of the equivalent capacity in the measuring circuit can vary the shapes and positions of the clusters on the classification map. These variations can be due to the process of manufacturing the test objects or by the fact of perform measurements in an environment where noise levels are different in nature and magnitude than those found in more controlled environments. Therefore, it is very important to properly select the separation intervals depending on the scenario to obtain a clear characterization of all sources present during the measurements.

**Figure 11:**
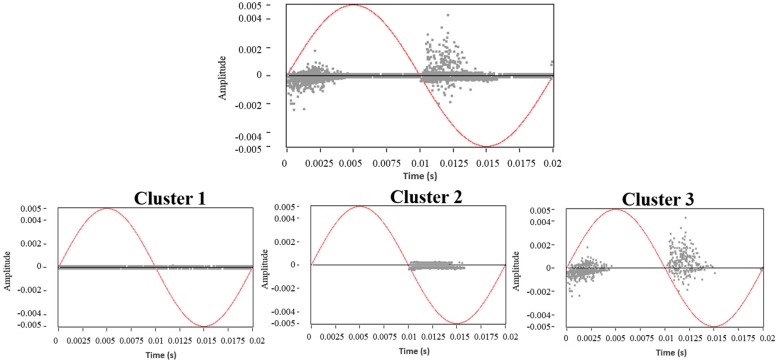
PRPD pattern for noise and PD simultaneously, **Up**: PRPD patterns for **Down**: cluster 1 (noise), cluster 2 (corona PD), and cluster 3 (internal PD).

The complete PRPD pattern, for the three PD sources acting simultaneously, is shown in Figure 11 (Up), where it is clear that PRPD interpretation seems to be quite complex even for an expert in the field. However, if the PRPD pattern for each cluster is represented individually, it can be clearly identify each of the sources present during the acquisition. As it can be seen in Figure 11 (Down), the PRPD associated to the cluster 1 represents the captured electrical noise during the acquisition (uncorrelated pulses in phase). The PRPD of the cluster 2 corresponds to the typical PRPD for corona discharges, where highly stable PD magnitudes are observed for the negative maxima of the applied voltage. Finally, when selecting the cluster 3 from the PR map provides a “clean” PRPD representation typical from internal PD, where the high-magnitude discharges occur in the phase positions where the voltage slope is maximum.

**Figure 12:**
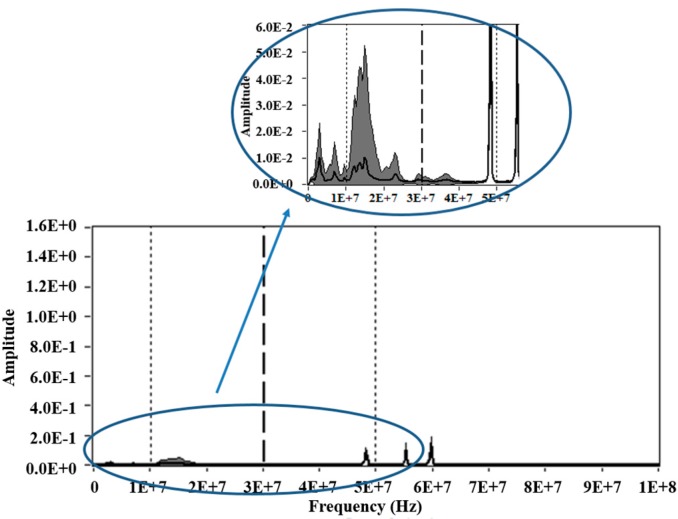
Average power spectrum density for PD and noise simultaneously measured.

Accordingly, when assessing the average power spectrum density of the pulses obtained in this experiment, it is observed that the selected intervals for PRL [10, 30] MHz and PRH [30, 50] MHz match bands where greater variability of spectral power occurs, that it is shown in the enlarged view in Figure 12. This justifies the separation obtained for each source on the PR map shown (see Figure 10).

**Figure 13:**
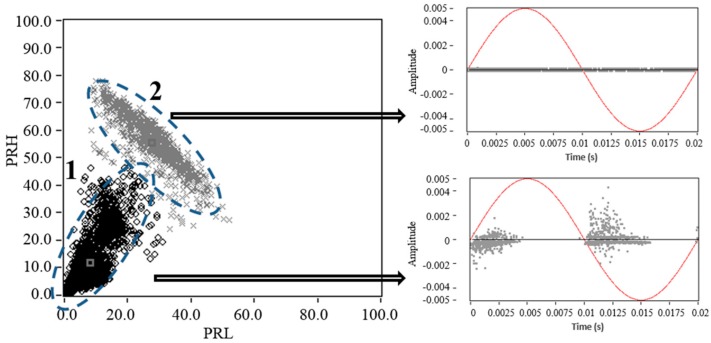
PR map for simultaneous PD (corona and internal) and noise activity. Frequency intervals for the power ratios calculations: *f_1L_* = 20 MHz, *f_2L_* = 40 MHz, *f_1H_* = 40 MHz, *f_2H_* = 60 MHz.

On the contrary, if the PRL and PRH had other bands, where the variability of spectral power was lower, the separation of the sources would be impossible. This can be demonstrated if the intervals [20, 40] MHz for PRL and [40, 60] MHz for PRH are used, for example. As shown in the PR map in Figure 13 (Left), using these new intervals of separation (including bands where the variability of spectral power is low), only two different clusters can be identify, which they are also very close each other. Therefore, two types of sources are superimposed in a single cluster, this can be checked representing the PRPD patterns for each cluster, see Figure 13 (Right). In the PR map of the Figure 13, the cluster 2 corresponds to the electrical noise, while the cluster 1 corresponds with the two types of the two remaining sources (corona and Internal PD), which are clearly overlapping.

**Figure 14:**
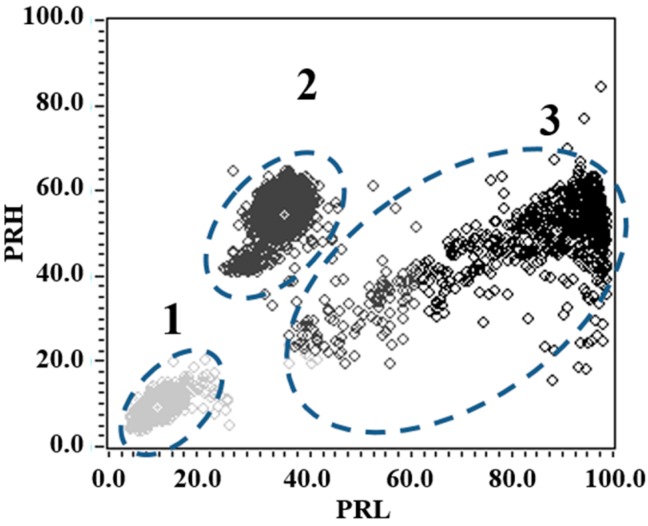
PR map for simultaneous PD and noise activity. Frequency intervals for the power ratios calculations: *f_1L_* = 2 MHz, *f_2L_* = 25 MHz, *f_1H_* = 15 MHz, *f_2H_* = 38 MHz.

Likewise, if other intervals, in which the frequency bands present greater variability of spectral power are selected, the sources separation will become more effective. For example, if *f_1L_* = 2 MHz, *f_2L_* =25 MHZ, *f_1H_* = 15 MHz, *f_2H_* = 38 MHz (*f_T_* = 60 MHz), intervals are used, a better separation between clusters is achieved (see Figure 14), compared with the separation obtained with the intervals used previously, see Figure 10 and Figure 13.

In this new separation map, the cluster 1 is associated with electrical noise pulses, cluster 2 with corona PD and cluster 3 with internal PD. Analysing the values of dispersion for PRL and PRH that are presented in Table 6, except *σ_PRL_* for corona PD cluster and *σ_PRH_* for internal PD, a clear decrease in the dispersion for each of the clusters using these new separation intervals it is shown. Additionally, a marked improvement in the *σ_PRL_/σ_PRH_* relation to the case of clusters associated with internal PD and electrical noise was achieved, since values close to 1 were obtained compared to the values shown in Table 5. For the cluster associated with corona PD, this relationship is increased, but not very significantly (1.35) compared to the previous value obtained in Table 5 (1.10).

**Table 6:** Results obtained of the standard deviation for each cluster associated with PD and noise, using *f_1L_* = 2 MHz, *f_2L_* =25 MHZ, *f_1H_* = 15 MHz, *f_2H_* = 38 MHz, *f_T_* = 60 MHz intervals.

Indicator	Noise	Corona PD	Internal PD
*σ_PRL_*	2.35	4.66	6.55
*σ_PRH_*	2.56	3.45	9.72
*σ_PRL_/σ_PRH_*	0.91	1.35	0.67

## 5. Conclusions

In this paper, the clustering capacity and the dispersion of the clusters obtained by the application of the SPCT to the pulses measured with a Rogowski coil have been studied. Results indicate that with this simple and inexpensive sensor, without magnetic core, the separation of different types of pulse sources using PR maps can be made adequately, even when there are several PD sources acting simultaneously.

Furthermore, this paper proposes using four different indicators, in order to find the separation intervals that allow a better separation of the PD sources and electrical noise present during the measurements. Three of these indicators (*σ_PRL_*, *σ_PRH_* and *σ_PRL_/σ_PRH_*) assist the operator of the classification tool to identify the intervals that enable to obtain those clouds of points more homogeneous. This allows an easier selection of the clusters on the PR maps for the further representation of the respective PRPD patterns. The fourth proposed indicator is based on a graphic tool that represents the average power spectrum density of the measured signals. This indicator allows selecting the bands of interest where the variability of the pulses is high and makes more feasible the separation between different groups of pulses. Analysing these indicators the manual selection of the PRL and PRH intervals can be easily improved, especially in those cases where the clusters obtained appear overlapped and/or there are suspicions of the presence of multiple sources during the measurements. The authors propose the use of these indicators, even when any other type of UHF sensor (ILS, HFCT, *etc*.) is employed.
